# Ultrasonographic Measurement of Muscle and Subcutaneous Fat Thickness for the Objective Assessment of the Nutritional Status of Alpacas

**DOI:** 10.3390/ani14243695

**Published:** 2024-12-20

**Authors:** Sonja Franz, Melanie Andrich, Thomas Wittek

**Affiliations:** Clinical Center for Ruminant and Camelid Medicine, Clinical Department for Farm Animals and Food System Science, University of Veterinary Medicine Vienna, 1210 Vienna, Austria; melanie.andrich@vetmeduni.ac.at (M.A.); thomas.wittek@vetmeduni.ac.at (T.W.)

**Keywords:** South American camelids, body condition score, body weight, body measurements, nutritional status

## Abstract

The aim of this study was to evaluate the ultrasonographic measurement of the subcutaneous fat and muscle layers at two different body locations as an objective tool with which to determine the nutritional status of alpacas. The results of ultrasonographic measurement were related to body weight, determined by scale, and body condition score (BCS), determined by a scoring system. Differences between gender (female/male) and different reproductive statuses (castrated/intact males, pregnant, and early or non-pregnant females) were evaluated. In total, 160 alpacas were examined. Ultrasonography was performed first at the lumbar region, positioning a linear probe (8 MHz) between the second and third lumbar vertebrae perpendicular to the spinal column, and in the gluteal region, measuring the distance between skin, the subcutaneous fat layer, and the muscle layer. The results showed that the gender and pregnancy status of females had a significant influence on the ultrasonographic measurements at both localizations. Significant associations were found between body weight and the BCS. The BCS and ultrasonographic-measured soft-tissue thicknesses at both localizations were significantly associated for males and early or non-pregnant females. According to these results, ultrasonography can be recommended as an objective method with which to determine the nutritional status of alpacas.

## Simple Summary

The determination of the nutritional status of alpacas is routinely performed via the inspection and palpation of different body regions following a body condition scoring system. Body condition scoring is a helpful and important tool with which to either detect chronic diseases at an early stage or to determine accurate dietary adjustments to a feeding program. As body condition scoring via visual appraisal and palpation is a subjective method, ultrasonographic measurement of back fat thickness is routinely used in cattle for the objective determination of nutritional status, which has not been described for alpacas so far. Consequently, it was the objective of this study to evaluate the ultrasonographic measurement of soft-tissue thickness at two different body sites in alpacas. The present study identified this technique as a reliable tool for the determination of nutritional status of male and early or non-pregnant alpacas; therefore, it can also be recommended in this animal species as an objective assessment method.

## 1. Introduction

The number of South American camelids (SACs), especially alpacas, is steadily increasing in Europe. Consequently, improvements in herd health management are required, resulting in challenges for SAC farmers and veterinarians [1,2].

The continuous assessment of the health and nutrition status of animals is an important part of herd health management that must be performed by animal handlers and veterinarians. The repeated assessment of body condition has been recommended as a routine measure, being an essential part of herd health plans to facilitate the early detection of pathological conditions [3,4,5,6,7]. Chronic diseases typically result in a loss of body condition [5,7]; in particular, infections with endoparasites are a common reason for weight loss and may lead to the death of an animal when diagnosed too late [8]. A proper body condition is also important for the adequate function of physiological metabolic processes and production. For instance, it is known that the body condition of dams has a positive association with the birth weight of crias [9], and crias with higher birth weights show higher survival rates [9,10,11]. Furthermore, associations between body condition, fleece growth rate, and fiber diameter have been described [12,13].

Visual appraisal for body condition scoring, which is a routine procedure in cattle [14], is not recommended in SACs due to the dense fleece (unless the animals have very recently been shorn) [4,7,15]. This also applies to small ruminant breeds carrying substantial amounts of wool in which the palpation of specific body regions is essential for body condition scoring [16,17,18,19,20]. To the best of our knowledge, the first description of body condition scoring in SACs was published by Johnson in 1994 [21]. Alongside this, several methods have been described that are based on the palpatory examination of different body parts to assess muscle and fat layers [4,7,12,22,23]. However, although the diagnostic value of the palpation of the muscles and subcutaneous fat is superior to visual appraisal, the method is still subjective to a certain degree, and readings may substantially vary between examiners [4,6].

To obtain more objectivity, the measurement of body weight by using a scale is an alternative [15,24]; however, because of differences in body size, body confirmation, the growth of the fetus, the increase in placenta weight and fetal fluid volume during pregnancy, or even the impact of feed and water intake, the determination of body weight is influenced by several parameters and therefore cannot be put on the same level as body condition.

The ultrasonographic measurement of back fat thickness to assess nutritional status has been developed in cattle as a method with improved objectivity, and it is widely used as a routine technique in cattle practice [25,26,27,28,29,30]. In cattle, the thickness of skin and back fat is measured sonographically in the gluteal region by using the deep body fascia as a landmark. In cattle, muscle thickness is not included in the measurements [27,28].

Ultrasonographic measurements with which to estimate body conditions have also been described for small ruminants; however, in contrast to cattle, not only fat but muscle layer thickness is also included in the measurements [31]. In sheep and goats, the ultrasonographic measurement of rump fat thickness (RFT) and sternal fat thickness (SFT) is frequently used [20]. The probe is either placed between the third and fourth lumbar vertebrae on the lumbar muscle (RFT) or over the third sternebra of the sternum (SFT), and soft-tissue thickness (skin, fat, and muscle) is measured; however, alternative measurement procedures and body locations have been used to estimate body condition [31,32].

Using literature search engines such as Scopus, Web of Science, Google Scholar, and PubMed, the authors found no reports on the ultrasonographic measurements of fat and muscle layers as an objective method for determining the nutritional status of SACs. The first objective of this study is to apply ultrasonography to measure the thickness of the subcutaneous fat and muscle layers at different body sites. The second objective is to associate ultrasonographic measurements with body weight and body condition score depending on gender and reproductive status.

The hypothesis of this study is that the ultrasonographic measurements of soft-tissue thickness (skin, subcutaneous fat, and muscle) are closely associated with body condition score and body weight, and can therefore be used as an objective assessment method.

## 2. Materials and Methods

### 2.1. Animals

This prospective cross-sectional study was performed on alpacas housed in different farms in Austria between September 2021 and May 2022. A convenience sample of alpacas older than 1 year were included in this study.

Animal species (Huacaya alpaca/Suri alpaca), age, gender (male/female), and pregnancy status of females (pregnant more than 6 months/non-pregnant or 6 or fewer months pregnant) or reproductive status of males (intact males/castrated alpacas) were documented. Further on, a classification as to whether the fleeces of alpacas were shorn or not (fleece length < 2 cm as suggested by Navarre et al. [33] and Heath et al. [34]) was performed. The differentiation of pregnancy status of females was used since substantial growth of the fetus starts in the final trimester of pregnancy [35].

All examinations were performed on a standing animal. The animal was held with only a halter and a rope by their owner. In all animals the body condition score was always assessed by the same person (experienced in clinical SAC work), followed by the ultrasonographic measurement of the subcutaneous fat and muscle layers at two different body regions. The body weight of the animal was determined by using a scale.

### 2.2. Body Condition Score

The body condition score (BCS) of each alpaca was calculated as a mean value by using scores for visual appraisal and palpation in 4 different body regions: the visual appraisal of the proximal front and hind limbs as well as sternal region, and the palpation of the rib cage and the lumbar region. A scoring system from 1 to 5 was used. A score of 1 indicated emaciated animals (no fat and a minimal amount of muscle), a score of 3 was considered physiological, and a score of 5 stood for overconditioned animals with substantial subcutaneous fat depots [36].

### 2.3. Ultrasonographic Examination

The ultrasonographic examination of non-sedated alpacas was, as mentioned before, performed on a standing animal, and no restraint chute was used. A portable ultrasonographic machine (DP-50 Expert^®^, Mindray Medical Germany GmbH, Darmstadt, Germany) with a linear probe (59 mm × 10 mm, 8 MHz) was used. In all animals, the ultrasonographic examination was performed on the left side of the body. The fleece in the area of interest was parted using fingers, regardless if the animal was shorn or not, and alcohol (70% ethanol) was used as the contact medium between the probe and skin. Ultrasonographic measurements were performed in a 2D modus by using the measuring advice of the ultrasound machine.

**Figure 1:**
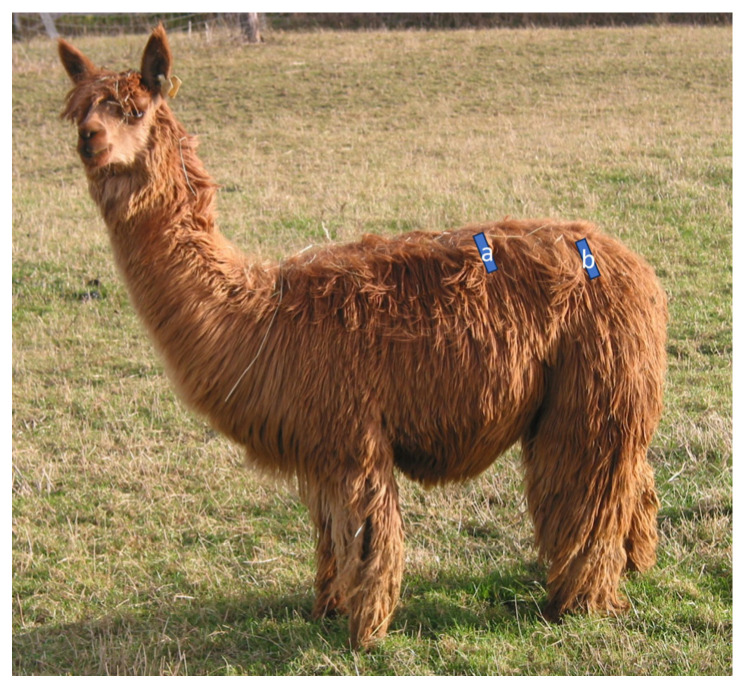
Position of probe for the ultrasonographic measurement of soft-tissue thickness (skin, fat, and muscle) in the lumbar (US-L) region (a) and in the gluteal (US-G) region (b) in an alpaca.

**Figure 2:**
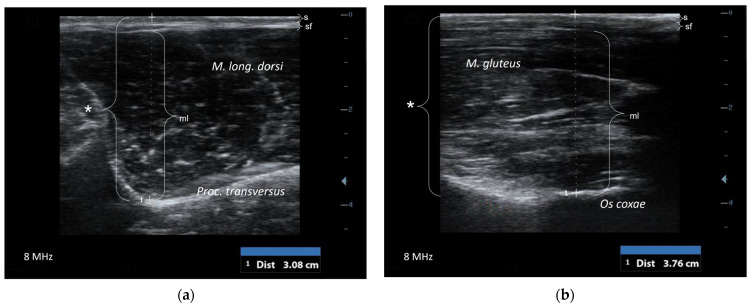
Transversal scan (**a**) of the lumbar region (US-L) between the first and second lumbar vertebrae: the distance (*) between the skin (s), subcutaneous fat (sf), and muscle layer (ml) was measured by using an ultrasonographic measuring device. The soft tissue is framed by the bony-structured transverse process of vertebrae. Transversal scan (**b**) of the gluteal region (US-G): the distance (*) between the skin (s), subcutaneous fat (sf), and muscle layer (ml) was measured. The muscle is framed by the bony structure of the pelvic bone.

The probe was positioned in a transversal position at two different regions: the first localization was in the lumbar region (US-L) between the first and second lumbar vertebrae perpendicular to the lumbar spine (Figure 1a). In this region the soft tissue is framed by the L-shaped bony structure formed by the spinous and transverse process of vertebrae. The ultrasonographic measurement of the thickness of soft tissue included the skin, subcutaneous fat, and muscle (*M. longissimus dorsi*), as shown in Figure 2a.

The second location for ultrasonographic measurement was the gluteal region (US-G), as has been described by Schröder and Staufenbiel in 2006 [27] for cattle. The ultrasound probe was placed on a line between coxal tuberosity and ischial tuberosity. The distance of that line was divided into five equal parts; the probe was placed on the transition between the second caudal and the fifth caudal (Figure 1b). The distance between skin, subcutaneous fat, and the gluteal muscle layer, which is ventrally bordered by the bony structure of the pelvic bone, was measured via ultrasonography (Figure 2b).

### 2.4. Body Weight

The body weight (BW) was measured by using a calibrated portable scale (PS1000^®^, Brecknell, Fairmont, MN, USA). The scale was covered with a thin rubber mattress to reduce the risk of skidding and increase the acceptance by the animals.

### 2.5. Statistical Analysis

The data were recorded and stored in an Excel table (Microsoft Excel^®^, version 2310, build 16.0.16924.20054, Microsoft Corporation, Redmont, WA, USA); calculations of the arithmetic mean (AM), standard deviation (SD), and coefficient of variation (CV) were also performed by using the Excel spreadsheet. For all other statistical procedures, SPSS 27.0 was used. Levene’s test, assessing the equality of variances for the variables, showed that the data followed a normal distribution. Stepwise regression analysis was performed to identify parameters which have significant effects on the ultrasonographic measurements. An ANOVA was used to compare different groups (advanced pregnant females, early or non-pregnant females, and intact and castrated males). Pearson correlation coefficients were calculated between the parameters BCS, body weight, and soft-tissue thickness of the two locations, US-L and US-G. *p*-values below 0.05 indicated statistical significance.

This study was discussed and approved by the institutional ethics and animal welfare committee in accordance with GSP guidelines and national legislation (ETK 009/01/2021).

## 3. Results

The study was performed on 160 alpacas (Huacaya alpaca (n = 154), Suri alpaca (n = 6)) older than 1 year housed in eight different herds in Austria. The oldest alpaca examined was 19.5 years (median age: 6.2 years). Seventy-nine of these were male alpacas, with a smaller number of castrated animals (n = 23). Among the 81 female alpacas were 44 alpaca mares that were pregnant for more than 6 months and 37 alpacas that were non-pregnant or less than 6 months pregnant. Only a small part of the alpacas had been recently shorn (n = 39), while 121 alpacas were not shorn at the time of examination. The detailed distribution of male and female alpacas, according to their reproductive status, is shown in Table 1.

The complete set of measurements (determination of BCS, ultrasonographic measurement of soft-tissue thickness, and body weight by scale) was successfully performed in 154 animals (Table 2). The body weight could not be measured in three animals due to technical problems of the scale, and in another three animals the ultrasonographic measurements in the gluteal region could not be performed due to lacking cooperation of the animals (Table 2). All other alpacas tolerated the whole procedure without any complications; ultrasonography of both regions was performed within 10–30 s.

In both regions (the lumbar and gluteal regions) the bony structures (US-L: transverse process of vetebrae; US-G: pelvic bone) were used as landmarks, allowing for standardized ultrasonographic examination. The intra-examiner variability in ultrasonography measurements was characterized via a variation coefficient of 0.04 ± 0.005 for US-L and one of 0.05 ± 0.007 for US-G. The inter-examiner variation coefficient was 0.03 ± 0.004 for US-L and 0.07 ± 0.009 for US-G.

The regression analysis showed that gender (r = 0.56, *p* < 0.01) and the pregnancy status of females (r = 0.35, *p* < 0.05) had a significant influence on the ultrasonographic measurements at both localizations (Table 3 and Table 4). In contrast, there was no significant influence as to whether the males were intact or neutered (*p* = 0.35) and if the animals had recently been shorn or not (*p* = 0.69). Since there was no difference between castrated and intact males, the males are presented as one group. Male animals had a significantly higher body weight and BCS in comparison to female alpacas. Significant moderate-to-strong associations were found between body weight and BCS in addition to body weight and ultrasonographic-measured soft-tissue thickness at the two localizations. The BCS and ultrasonographic-measured soft-tissue thickness at both localizations were significantly associated for male animals and early or non-pregnant females. In contrast, the associations of the parameters were generally weaker and partially statistically not significant in pregnant females with a pregnancy longer than 6 months (Table 3 and Table 4).

The arithmetic means and SDs for US-L and US-G soft-tissue thickness measurements are separately provided for the body condition classes (scores of 1 to 5) in male animals and in females that were not pregnant or early pregnant (Table 5). The range of ultrasonographic measurements of soft-tissue thickness in animals with a BCS of 3 to <4 are considered the most desirable results.

## 4. Discussion

Currently, the methods for assessing the nutritional status of animals are determining body weight using scales and assessing the body condition score via visual appraisal and palpation of an animal at different regions [7,15].

The measurement of body weight by using scales has been recommended in SACs as part of herd health management plans [24], but in numerous herds there are either no scales available or the owner finds it impractical to train the animals to become used to a scale [37]. Furthermore, body weight is influenced by feed and water intake, as well as the amount of fleece carried by animals. Additionally, pregnancy status has an impact on body weight.

Although guidelines have been developed to increase the objectivity of the assessment of body condition, scoring is still a subjective procedure with some variability between examiners [6]. In cattle, it has been reported that there is a tendency for examiners to estimate towards the average values and avoid extreme scores [27]. In this regard, there is no information for SACs, but it seems to be analogous to that for cattle.

However, in contrast to measuring body weight by using a scale, the BCS is not influenced by short-term events like food or water intake. Body condition scoring in SACs is well established, and different scoring systems are available [36]; however, due to the dense fleece palpation of different body regions for determination of body condition score is superior to visual assessment [7].

The aim of the study was to apply an objective method such as ultrasonography for measuring the thickness of subcutaneous fat and muscle layers at two body sites and to evaluate the correlation between the results of ultrasonographic measurements, the BCS, and body weight determined by a scale. This study showed that the method of ultrasonographic soft-tissue thickness measurement result in precise and reproducible measurements, indicated by the low inter- and intra-examiner variability. The high repeatability of ultrasonographic measurements of soft-tissue thickness in SACs is like what has been reported for sheep [20] and cattle [29]. One limitation of this study is that the examiner performing the ultrasonographic examination could not be blinded to the body condition of the animal, since it is unavoidable to palpate the animal when placing the ultrasound probe.

The ultrasonographic examination method does not require clipping hair and does not take longer than 30 s, which may increase its feasibility and acceptance in practice. Only three animals did not tolerate the ultrasonographic examination in the gluteal region, but all examinations could be performed safely in the lumbar region.

The measurement of soft-tissue thickness in alpacas is more like the technique which is used in sheep [20,38] than the procedure used in cattle [27]. The present study showed that the deep fascia (*Fascia trunci profunda*), which is the landmark in cattle in the gluteal region, could not be visualized in alpacas. In contrast with sheep and cattle, alpacas only have minor subcutaneous fat depots in this region. The soft-tissue thickness in alpacas was defined as the distance between the skin, subcutaneous fat, and gluteal muscle. The pelvic bone in US-G could be identified in all animals and used as a landmark for measuring. The measurement of soft tissue was also performed sonographically in the lumbar region, because this is the region where the palpation of the muscle layer is performed to estimate the body condition score [7]. The same equipment was used for ultrasonographic examination and allowed for the excellent visualization of the skin, subcutaneous fat, and muscle. Additionally, in this region the subcutaneous fat layer was very thin. The bony structure displaying the transverse process of vertebrae was a helpful anatomical landmark for probe positioning. The results of ultrasonography in both regions were well correlated except for the group of females in later pregnancy. As both regions can be considered equally suitable, the choice is left to the examiner’s preference. The associations between ultrasonographic-measured soft-tissue thickness in both regions and the BCS as well as body weight were substantial for male and early or non-pregnant females. The associations were much weaker for females in advanced pregnancy, such that we cannot recommend ultrasonography for the assessment of the BCS or body weight in this group of animals. It might be possible to improve the diagnostic value by including other biometric parameters, which needs to be explored. In contrast, the soft-tissue thickness showed high correlation with the body condition for males and for early or non-pregnant females, allowing for the recommendation of ultrasonography for the objective assessment of nutritional status.

## 5. Conclusions

Although this study is based on a substantial number of animals, the recommended ranges need to be evaluated on more animals and with a multicentric approach, including a higher number of examiners and animals. In particular, the number of animals with a very low and a very high BCS is rather limited.

The hypothesis of this study could be confirmed: that ultrasonography can be used to determine soft-tissue thickness (skin, subcutaneous fat, and muscle), which is closely associated with the body condition score and body weight. The results of this study demonstrate the potential of ultrasonography as an objective assessment method of nutritional status in alpacas and therefore can be recommended for vets as additional tool for managing herd health when performed on a regular base.

## Figures and Tables

**Table 1:** Number of alpacas (n = 160) with distribution concerning gender (male/female) and pregnancy (females) as well as reproductive (males) status.

	Male		Female	
Alpacas	Intact	Castrated	Pregnant > 6 Months	Non-Pregnant or Pregnant < 6 Months
n	56	23	44	37
%	35.00	14.38	27.50	23.12

**Table 2:** Results of body weight (BW), body condition score (BCS), and measurement of ultrasonographic soft-tissue thickness in the lumbar (US-L) and gluteal region (US-G) in alpacas given as the arithmetic mean (AM), standard deviation (SD), maximum (MAX), and minimum (MIN).

Measured Parameter	N *	AM	SD	MAX	MIN
BW (kg)	157	65.60	14.26	106.60	24.50
BCS	160	2.84	0.70	4.50	1.25
US-L (cm)	160	3.90	0.66	5.75	2.10
US-G (cm)	157	4.09	0.65	5.86	2.04

* N = number of animals.

**Table 3:** Body weight (BW), body condition score (BCS), and soft-tissue thickness in the lumbar (US-L) and gluteal regions (US-G) for male and female alpacas given as the arithmetic mean (AM) and standard deviation (SD). Different alphabetical indices (a, b) indicate statistical differences between males and females.

Measured Parameter	Gender	N *	AM	SD
BW (kg)	Male	78	67.63 a	14.98
	Female	79	63.57 b	13.30
BCS	Male	79	3.01 a	0.69
	Female	81	2.68 b	0.68
US-L (cm)	Male	76	4.04 a	0.72
	Female	78	3.75 b	0.58
US-G (cm)	Male	75	4.23 a	0.68
	Female	75	3.96 b	0.60

* N = number of animals.

**Table 4:** Associations between body weight (BW), body condition score (BCS), and soft-tissue thickness measured sonographically in the lumbar region (US-L) and in the gluteal region (US-G). The data are given for the groups: all animals, males, females in advanced pregnancy (>6 months pregnant), and females that are early or non-pregnant (<6 months pregnant).

Alpacas	Correlation Coefficients (*p*-Value) Between Measured Parameters					
	BW:BCS	BW:US-L	BW:US-G	BCS:US-L	BCS:US-G	US-L:US-G
All alpacas (n = 160)	0.46 (<0.05)	0.63 (<0.001)	0.71 (<0.001)	0.77 (<0.001)	0.58 (<0.01)	0.77 (<0.001)
Males (n = 79)	0.59 (<0.001)	0.76 (<0.001)	0.84 (<0.001)	0.77 (<0.001)	0.72 (<0.001)	0.86 (<0.001)
Females pregnant > 6 months (n = 44)	0.12 (0.43)	0.39 (<0.01)	0.40 (<0.01)	0.70 (<0.001)	0.25 (0.12)	0.53 (<0.001)
Females non-pregnant or pregnant < 6 months (n = 37)	0.43 (<0.001)	0.75 (<0.001)	0,73 (<0.001)	0.85 (<0.001)	0.78 (<0.001)	0.89 (<0.001)

n = number of animals.

**Table 5:** Arithmetic mean (AM) and standard deviation (SD) of lumbar soft-tissue thickness (US-L) and gluteal soft-tissue thickness (US-G) for different body condition score (BCS) classes; different alphabetical indices (a, b, c and d) indicate statistical differences between males, and different numerical indices (1, 2, and 3) indicate statistical differences between the early or non-pregnant females (<6 months pregnant).

BCS	Gender	N *	US-L (cm): AM ± SD	US-G (cm): AM ± SD
1 to <2	Male	5	2.50 ± 0.34 (a)	2.86 ± 0.58 (a)
	Females non-pregnant or pregnant < 6 months	7	2.80 ± 0.49 (1)	3.13 ± 0.46 (1)
2 to <3	Male	30	3.80 ± 0.57 (b)	3.99 ± 0.49 (b)
	Females non-pregnant or pregnant < 6 months	17	3.57 ± 0.32 (2)	3.72 ± 0.41 (2)
3 to <4	Male	38	4.28 ± 0.54 (c)	4.44 ± 0.48 (c)
	Females non-pregnant or pregnant < 6 months	12	4.24 ± 0.49 (3)	4.34 ±0.52 (3)
4 to 5	Male	6	4.89 ± 0.49 (c)	5.22 ± 0.62 (d)
	Females non-pregnant or pregnant < 6 months	1	4.84	5.07

* N = number of animals.

## Data Availability

The data that support the findings of this study are available from the corresponding author upon reasonable request.

## Abbreviations

AM	Arithmetic mean
BCS	Body condition score
BW	Body weight
CV	Coefficient of variation
MAX	Maximum
MIN	Minimum
US-G	Ultrasonography soft-tissue thickness gluteal region
US-L	Ultrasonography soft-tissue thickness lumbar region
RFT	Rump fat thickness
SACs	South American camelids
SD	Standard deviation
SFT	Sternal fat thickness
